# Correction: Vol. 30, No. 2

**DOI:** 10.3201/eid3108.C23108

**Published:** 2025-08

**Keywords:** errata, erratum, correction

The name of author Alexis M. Siegler was incorrect in Contribution of Limited Molecular Testing to Low Ehrlichiosis Diagnosis in High Incidence Area, North Carolina, USA (A.M. Siegler et al.). The article has been corrected online (https://wwwnc.cdc.gov/eid/article/31/2/24-0281_article).

